# Combined network analysis and machine learning allows the prediction of metabolic pathways from tomato metabolomics data

**DOI:** 10.1038/s42003-019-0440-4

**Published:** 2019-06-18

**Authors:** David Toubiana, Rami Puzis, Lingling Wen, Noga Sikron, Assylay Kurmanbayeva, Aigerim Soltabayeva, Maria del Mar Rubio Wilhelmi, Nir Sade, Aaron Fait, Moshe Sagi, Eduardo Blumwald, Yuval Elovici

**Affiliations:** 1 Department of Plant Sciences, University of California, Davis, CA USA; 2 Telekom Innovation Labs, Department of Software and Information Systems Engineering, Ben-Gurion University of the Negev, Beer Sheva, Israel; 3 French Associates Institute for Agriculture and Biotechnology of Drylands, Jacob Blaustein Institutes for Desert Research, Ben-Gurion University of the Negev, Sede Boqer, Israel; 4 School of Plant Sciences and Food Security, Tel Aviv University, Tel Aviv, Israel

**Keywords:** Network topology, Plant biotechnology, Machine learning, Computational models, Metabolomics

## Abstract

The identification and understanding of metabolic pathways is a key aspect in crop improvement and drug design. The common approach for their detection is based on gene annotation and ontology. Correlation-based network analysis, where metabolites are arranged into network formation, is used as a complentary tool. Here, we demonstrate the detection of metabolic pathways based on correlation-based network analysis combined with machine-learning techniques. Metabolites of known tomato pathways, non-tomato pathways, and random sets of metabolites were mapped as subgraphs onto metabolite correlation networks of the tomato pericarp. Network features were computed for each subgraph, generating a machine-learning model. The model predicted the presence of the β-alanine-degradation-I, tryptophan-degradation-VII-via-indole-3-pyruvate (yet unknown to plants), the β-alanine-biosynthesis-III, and the melibiose-degradation pathway, although melibiose was not part of the networks. In vivo assays validated the presence of the melibiose-degradation pathway. For the remaining pathways only some of the genes encoding regulatory enzymes were detected.

## Abstract (web summary)

David Toubiana et al. report the combination of correlation-based network analysis and machine-learning techniques to detect metabolic pathways from quantitative metabolic data. They demonstrate the utility of their method by studying metabolic pathways in tomato.

## Introduction

The reconstruction of metabolic pathways is a complex process based on a constraint-based bottom-up approach; such reconstruction typically uses gene annotation and ontology, computational derivation, and discrete manual curation, requiring a priori knowledge of the stoichiometry between compounds, thermodynamic information of the pathway’s reactome, as well as its cellular compartmentalization, and other factors. Due to the complexity of reconstruction, metabolic pathways are more often predicted computationally rather than on substantial experimental evidence^1^. The reconstruction of metabolic networks follows a defined set of steps; initiated at the known biochemistry, genomics, and physiology, followed by the governing of the physico-chemical constraints, proceeded by flux distribution predictions, and finalized by the determination of which of the offered solutions translate into meaningful physiological states^2,3^. Regardless of whether or not they are fully validated, metabolic pathways are collected in databases of genome-scale hypernetworks^4,5^, e.g., PlantCyc (http://www.plantcyc.org/)^6^, BioCyc (http://biocyc.org/)^7^, and KEGG (http://www.genome.jp/kegg/)^8^.

Complementary to the constraint-based approach, metabolite networks—constituted on high-throughput data metabolite profiles—provide an attractive method for studying the coordinated behavior of metabolites without the need for a priori knowledge. Metabolite profiles are correlated based on mathematically defined (dis)similarity measures^9^, which are subsequently transformed into network form, where nodes represent the metabolites and the links between them the correlation coefficients.

Metabolite correlation-based networks are often reconstructed based on the exploitation of the natural variability of mapping populations^10–14^ or collections of different varieties or cultivars^15–17^ as they provide a large sample size, which stabilizes the correlation and reduces the error rate. Correlation-based network analysis (CNA) explores the structural properties of graphs that can be used to interpret metabolite networks and even postulate hypotheses^18^. Nonetheless, although CNA and graph theory are equipped with a myriad of tools^19–21^, many studies limit themselves to employing CNA for the study of the global structure and relationships of metabolite data. For the current study, we exploit the tools from graph theory.

Machine learning (ML) employs a collection of techniques that allow computers to learn from existing data without being explicitly programmed^22^. An ML approach to predict metabolic pathways in bacteria has been proposed based on properties of metabolic pathways as defined in genome-scale networks^23^. Although various ML algorithms exist to tackle problems for studying metabolic profiles, the power of ML algorithms has been underutilized in the analysis of metabolic correlation networks.

In this study, we delved deep into the possibilities of CNA and ML by combining them to predict metabolic pathways in correlation networks in the pericarp of a tomato introgression line population. We demonstrate that this method can be essentially used for functional metabolomics. We do so by mapping existing metabolic pathways onto the metabolite correlation networks followed by the computation of a set of network properties for each pathway to derive an ML model. The resulting ML model was then used to predict the existence of yet unidentified pathways based on the mapping of pathways onto the correlation networks and computation of the same set of network properties. To validate the model, we applied several in vivo experiments on the positively predicted, yet unidentified pathways.

To the best of our knowledge this is the first study that employs structural analysis of metabolite correlation networks in order to identify metabolic pathways.

## Results

The identification of metabolic pathways is a key aspect in understanding the metabolism of an organism of interest. PlantCyc (http://www.plantcyc.org/) is a collection of metabolic pathways found in plants. TomatoCyc is the subset of PlantCyc containing metabolic pathways found in tomato—notwithstanding the possibility that some of the remaining PlantCyc pathways may also be found in tomato. The methods introduced in this paper facilitated the identification of previously unknown metabolic pathways within the tomato pericarp using supervised ML techniques combined with metabolite CNA. It does so based solely on reactions and may not be used to predict differences in catalytic activity.

Given a set of tomato pathways (positive instances) and a set of pathways that do not exist in tomato (negative instances), a supervised ML model was induced in order to classify any given pathway (test instance) as either tomato (positive) or non-tomato (negative). A set of numeric profiles (feature vectors) of positive and negative instances (the training set) was utilized by ML algorithms during the training phase in order to induce such a model.

**Fig. 1:**
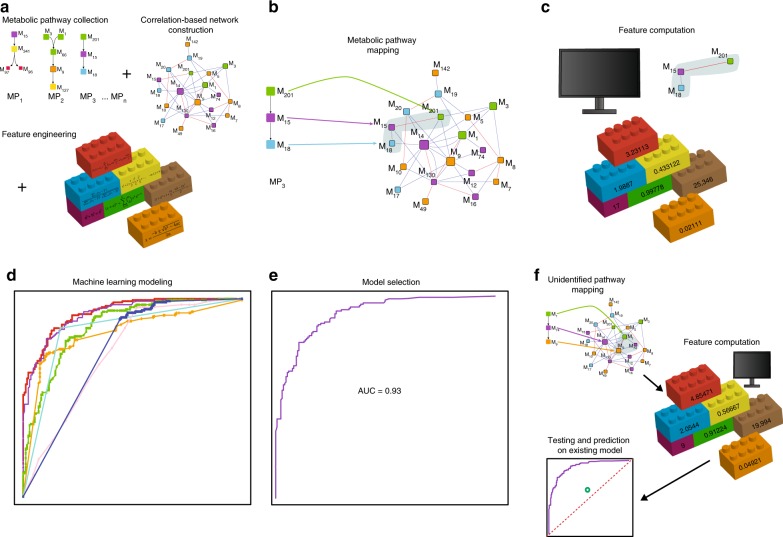
Combined correlation-based network analysis and machine learning workflow. The workflow of the current study: **a** Metabolic pathways were gathered from existing repositories. In parallel, correlation-based networks of metabolites were constructed for the tissue of the organism of interest (here, the tomato pericarp). In addition, a vector of features was engineered based on network properties. **b** Metabolic pathways with partial to full coverage in the correlation networks were mapped to the networks. Each pathway was considered as a single instance. Training and test sets were proposed based on the existence of the pathways in the tomato. **c** A set of features was computed for each instance in the training set (for the current study 148 * 3 networks = 444 features in total). **d** The training set was used to generate different ML models. **e** The model that generated the best performance measures (the AUC) was selected. The ML model was validated in silico using cross-validation. **f** Test set instances were mapped onto the networks with subsequent feature computation. The proposed ML model was used to predict the potential existence of unidentified pathways in the tomato pericarp

The numeric profiles of metabolic pathways were computed from tomato CNs based on the tomato introgression line mapping population^24^ as presented in Toubiana et al.^11^. The original dataset^10^ contained metabolic profiles of the central metabolism of the tomato fruit for three different harvesting seasons, hereinafter referred to as seasons I, II, and III. For each season, a weighted, undirected CN was constructed. Network links were weighted according to their correlation coefficient, allowing negative values. The CN for season I included 75 nodes, corresponding to the 75 metabolites, and 473 links; the CN for the season II was composed of 75 nodes and 869 links, while the CN for season III had 78 nodes and 338 links. Each pathway analyzed (train or test) was represented as a group of nodes in each one of the three CNs. A numeric profile was computed for each group of nodes in each CN (for details see the Methods section). Pathways that were part of the PlantCyc and MetaCyc (https://metacyc.org/) collections but not found in TomatoCyc were used to train and induce ML models. A workflow of the current study is presented in Fig. 1.

### Mapping identified plant and non-plant metabolic pathways

In total, the three seasons and the corresponding CNs contained 109 different metabolites, i.e., nodes, while 52 common metabolites were contained in all three CNs. Out of the 589 metabolic pathways listed in TomatoCyc, 169 pathways were identified to be mapped as a subgraph onto the three CNs. The mapping was partial in a sense that it allowed omitting compounds from the pathways that were not found in the 52 common metabolites. In other words, at least two compounds of a given pathway needed to intersect with the common set of 52 metabolites in order to be considered for pathway mapping. Consequently, only the pathway’s corresponding compounds were mapped followed by feature computation.

The superpathway of lysine, threonine, and methionine biosynthesis II, had 36% of its compounds within the networks, which resulted in the largest of all subgraphs. In total, 67 pathways were represented by exactly two compounds, while for three pathways all of their compounds were found in the CNs. The same analysis was repeated for the remaining 625 non-tomato plant pathways, identifying 33 pathways that shared at least two compounds with the tomato metabolite CNs. For the non-plant MetaCyc pathways, 151 pathways were identified that shared at least two or more compounds with the CNs. In both cases (tomato and non-tomato MPs), the largest number of compounds shared with the CNs was 18. Supplementary Fig. 1 illustrates the distributions of the relative portion of the metabolites of the different MPs mapped to the CNs, revealing a right-skewed distribution for all three-Cyc datasets. For the pathways corresponding to the TomatoCyc dataset the largest relative frequency of ~25% was observed at approximately 40% coverage, while for the pathways corresponding to the remaining PlantCyc and MetaCyc datasets the peak was reached at approximately 20% relative coverage with ~22 and ~31% relative frequency, respectively. To compare the relative distributions of coverage, a two-sided Kolmogorov-Smirnov test was employed, revealing that the PlantCyc vis-à-vis the TomatoCyc and the PlantCyc vis-à-vis the MetaCyc distributions were statistically equal (p-values 0.09681 and 0.09887, respectively), while the TomatoCyc vis-à-vis the MetaCyc distribution was significantly different (*p*-value 2.631e-06).

### ML model achieved high accuracy in classifying known pathways

The aforementioned 169 tomato pathways were used as the positive instances in the training set (Supplementary Data 1). Half of the negative instances (85) for training the ML classifier were randomly chosen from the 151 MetaCyc pathways. The second half was comprised of 85 random subsets (negative sampling) of the 52 common metabolites. The aforementioned 33 non-tomato plant pathways were not included in the training set (Supplementary Data 2).

We used 10-fold cross-validation to choose the best ML algorithm for the pathway classification problem and tune its parameters. There are multiple performance measures to evaluate the quality of ML models, including the area under the receiver operating characteristic curve (AUC), which is often used as the pivotal measure.

**Fig. 2:**
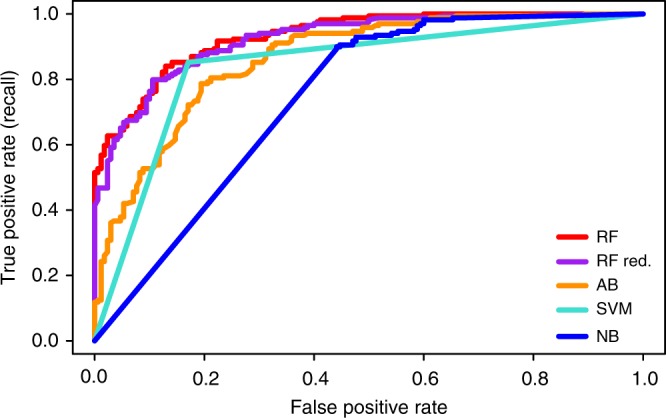
The receiver operating characteristic (ROC) curves. The figure shows the curves of the ROC for the ML models for the positive and negative class instances. Abbreviations within the figure represent the different ML algorithms: *RF* random forest, *RF red* random forest with reduced feature set, *AB* AdaBoost, *RT* random tree, *SVM* support vector machine, *NB* naïve Bayes

We applied various classifier algorithms (Fig. 2) and also created models with different feature combinations, i.e., models for each season individually, a season average model, and a model for all season features combined (Table 1). The random forest algorithm for all seasons combined rendered the best result, achieving an AUC of .932 and accuracy of 83.78% (284 correctly vs. 55 incorrectly classified instances, Supplementary Table 1). The random forest algorithm is an ensemble of generated decision trees for which the average prediction of the individual trees is produced^25^.

**Table 1:** Random forest model performance measure summary

	Class	True positive rate (Recall)	False positive rate	Precision	F-measure	AUC
All season features—model I	TomatoCyc pathways	0.917	0.241	0.791	0.849	0.932
	MetaCyc and random pathways	0.759	0.083	0.902	0.824	0.932
	Weighted average	0.838	0.162	0.847	0.837	0.932
Season I features—model II	TomatoCyc pathways	0.864	0.182	0.825	0.844	0.918
	MetaCyc and random pathways	0.818	0.136	0.858	0.837	0.917
	Weighted average	0.841	0.159	0.841	0.841	0.917
Season II features—model III	TomatoCyc pathways	0.876	0.229	0.791	0.831	0.91
	MetaCyc and random pathways	0.771	0.124	0.862	0.814	0.91
	Weighted average	0.823	0.177	0.827	0.823	0.91
Season III features—model IV	TomatoCyc pathways	0.828	0.306	0.729	0.776	0.876
	MetaCyc and random pathways	0.694	0.172	0.803	0.744	0.876
	Weighted average	0.761	0.239	0.766	0.76	0.876
Averaged seasons feature—model V	TomatoCyc pathways	0.858	0.212	0.801	0.829	0.914
	MetaCyc and random pathways	0.788	0.142	0.848	0.817	0.914
	Weighted average	0.823	0.177	0.825	0.823	0.914
Reduced features based on model I–model VI	TomatoCyc pathways	0.858	0.188	0.819	0.838	0.923
	MetaCyc and random pathways	0.812	0.142	0.852	0.831	0.923
	Weighted average	0.835	0.165	0.836	0.835	0.923

### Season II was identified as the main feature contributor

**Fig. 3:**
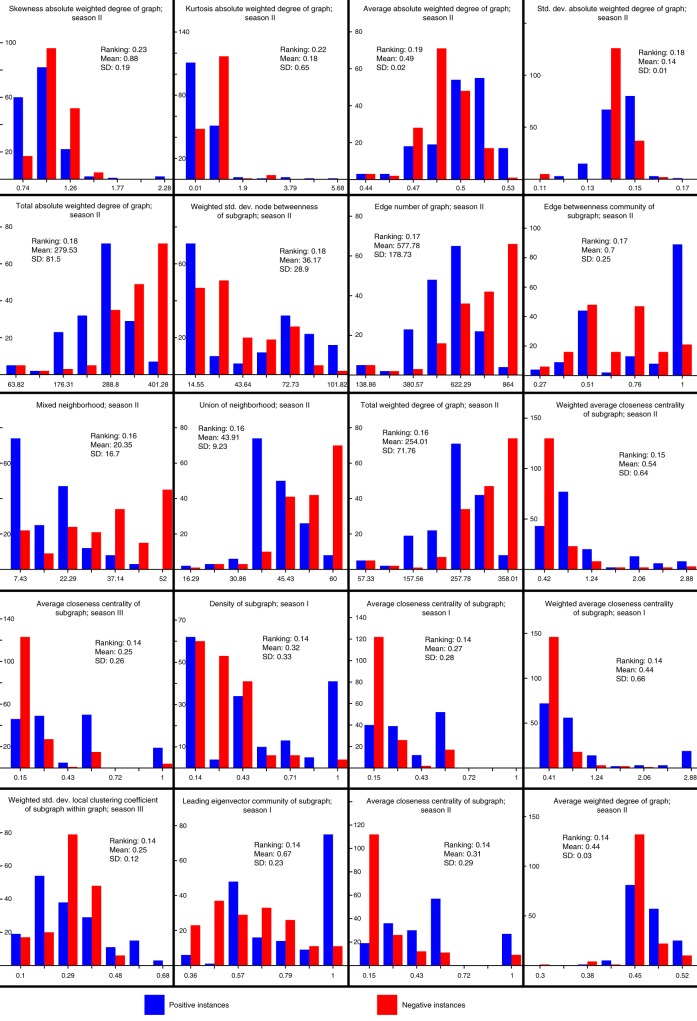
Top-20 ranked features; positive vs. negative instance distribution An attribute information gain algorithm was applied, ranking the contribution of the different features to the random forest model. The top-20 seeded features illustrated here were chosen to generate a reduced feature-set random forest model. The different graphs corresponding to the 20 features show the distribution of the computed features of the positive test set instances (blue) vs. the negative instances (red). X-axes represent values corresponding to features, y-axes represent instance counts

In order to identify the most contributing features and reduce potential overfitting, the features were evaluated using InfoGain^26^. Figure 3 presents the 20 top-seeded features used to reestablish an random forest model closest to the all-feature-model in terms of ML performance measures, while Supplementary Table 2 lists their definitions (a full ranking of the features is listed in Supplementary Data 3). For the 20-feature-model, accuracy of 83.48% was achieved with 283 correctly and 56 incorrectly classified instances. The AUC was estimated at 0.923, compared to the AUC of 0.932 of the all-feature-model (Table 1, Fig. 2).

We observed that the 12 highest ranked features (out of the 20-top seeded features) (Fig. 3) corresponded to season II. Nine of the 20 features represented network properties that described how well connected a node or a group of nodes was (see features 1-5, 11, 14, and 20 in Fig. 3 and Supplementary Table 2), revealing that nodes of tomato pathways maintained greater connectivity to each other than nodes of non-tomato pathways and of non-pathways (random subsets of metabolites). In particular, the distribution of positive (tomato pathways – blue) vs. negative (non-tomato pathways – red) instances in the density-of-subgraph-season_I feature emphasized this behavior.

Network centrality properties measure the importance of a node or link for maintaining the cohesiveness of a network. Here, the distribution of the centrality-related features (see features 12, 13, 15, 16, and 20 in Fig. 3 and Supplementary Table 2) highlighted that the metabolites of the negative instances were less central than the metabolites of the positive instances.

Community detection algorithms are applied to networks in order to elucidate the macroscopic structure of the network. Several different community detection algorithms have been postulated and successfully applied^20^. The distributions of features associated with the community structure (see features 8 and 18 in Fig. 3 and Supplementary Table 2) demonstrated that nodes in the CN associated with the tomato pathways tend to be grouped into the same community, in contrast to the nodes corresponding to the non-tomato pathways. For definitions of all features we refer the reader to the Methods section and Supplementary Data 4.

The 20-feature-set ML model was verified applying leave-one-out cross-validation (see Methods for more details and Supplementary Data 5), during which 84.62% of the 169 tomato pathways were classified correctly. When compared to the classification of millions of random subsets of metabolites, the prediction values of all tomato pathways fell within the first percentile (Supplementary Fig. 2), while the prediction values of 170 non-tomato pathways were, on average, (0.189) within the first quintile (Supplementary Fig. 2). As such, the leave-one-out cross-validation method validated the proposed random forest model.

### Classification of test set predicted 22 pathways in tomato

**Fig. 4:**
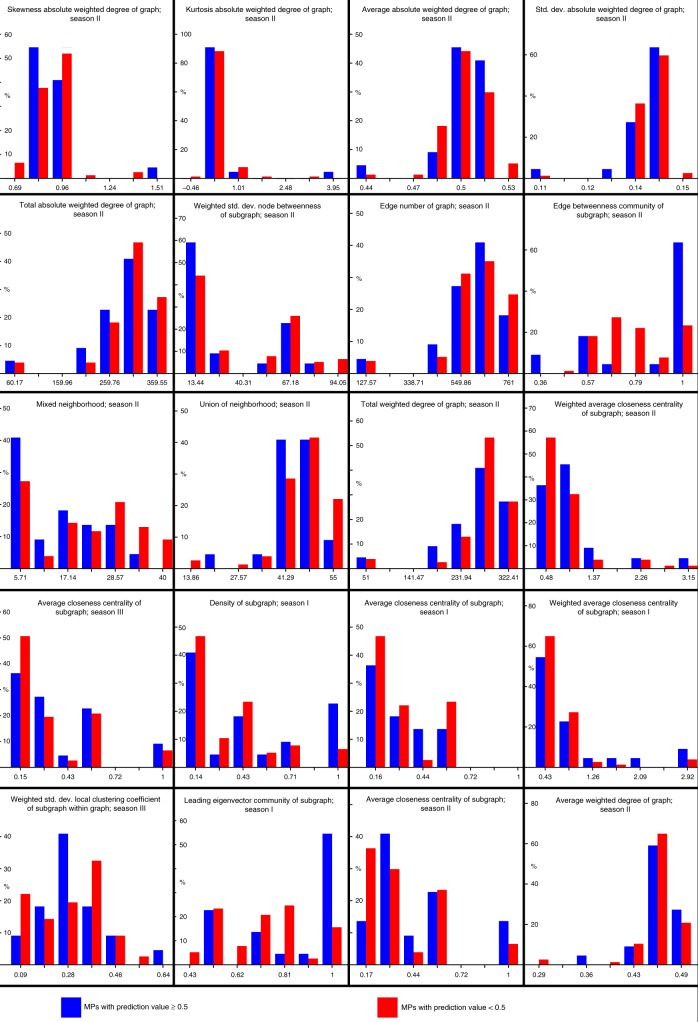
Top-20 ranked features; positively vs. negatively predicted metabolic pathways relative distribution The top-20 seeded features of the final ML model are illustrated to demonstrate show the distribution of the the positively (blue) vs. the negatively (red) predicted metabolic pathways relative distribution. X-axes represent values corresponding to features, y-axes represent relative instance counts

After validation, the abovementioned 33 plant pathways and the remaining 66 MetaCyc pathways that were not included in the training set, were classified by the trained ML model. Prediction values associated with these instances ranged from 0 to 1. Here, a prediction value threshold of 0.5 was chosen to forecast the potential existence of a pathway in the tomato (Table 2 lists all of the pathways with a prediction value ≥ 0.5, Supplementary Data 6 lists all of the pathways). In total, 22 pathways obtained a prediction value of 0.5 or greater, of which six were associated with PlantCyc pathways and 16 with MetaCyc pathways. The β-alanine degradation I pathway achieved the highest prediction value of 0.89. For the PlantCyc pathways, the melibiose degradation pathway achieved the highest prediction value of 0.68. While the inspection of the relative distribution of the 20 features revealed many differences between positively and negatively predicted metabolic pathways, three features emphasized the difference in particular (Fig. 4): the edge betweenness community of subgraph of season II showed higher values for the majority of the positively predicted metabolic pathways, indicating a greater edge betweenness for their corresponding subgraphs; for the weighted standard deviation local clustering coefficient of subgraph within graph feature of season III positively predicted metabolic pathways demonstrated a normal distribution, while negatively predicted metabolic pathways showed a bimodal, left-skewed distribution, suggestive for a greater variety of the local clustering coefficient of subgraphs of non-tomato predicted pathways; the leading eigenvector community of subgraph of season I illustrated a left-skewed distribution for the positively predicted metabolic pathways, showing that they tend to group themselves following a leading eigenvector community.

**Table 2:** Pathway existence prediction values for class 1

Database	Pathway	Original model	Sensitivity analysis average	Sensitivity analysis variance	Conform with original model average
MetaCyc	beta-alanine degradation I	0.89	0.631	0.01812	TRUE
MetaCyc	superpathway of butirocin biosynthesis	0.85	0.914	0.00990	TRUE
MetaCyc	isopenicillin N biosynthesis	0.85	0.879	0.01379	TRUE
MetaCyc	L-tryptophan degradation VII (via indole-3-pyruvate)	0.76	0.773	0.01815	TRUE
MetaCyc	L-tryptophan degradation IV (via indole-3-lactate)	0.76	0.843	0.01298	TRUE
MetaCyc	gliotoxin biosynthesis	0.75	0.843	0.01298	TRUE
MetaCyc	superpathway of scopolin and esculin biosynthesis	0.71	0.928	0.00850	TRUE
PlantCyc	melibiose degradation	0.68	0.534	0.08974	TRUE
PlantCyc	beta-alanine biosynthesis III	0.68	0.596	0.03190	TRUE
MetaCyc	apicidin F biosynthesis	0.68	0.862	0.01167	TRUE
MetaCyc	creatine biosynthesis	0.68	0.796	0.02079	TRUE
MetaCyc	mycolyl-arabinogalactan-peptidoglycan complex biosynthesis	0.65	0.708	0.02882	TRUE
PlantCyc	putrescine degradation I	0.63	0.749	0.02393	TRUE
PlantCyc	hypoglycin biosynthesis	0.61	0.824	0.01497	TRUE
MetaCyc	L-tryptophan degradation VIII (to tryptophol)	0.61	0.704	0.02038	TRUE
PlantCyc	lathyrine biosynthesis	0.6	0.639	0.02321	TRUE
MetaCyc	superpathway of L-methionine salvage and degradation	0.6	0.731	0.02034	TRUE
MetaCyc	superpathway of histidine, purine, and pyrimidine biosynthesis	0.58	0.481	0.03771	FALSE
MetaCyc	L-glutamate degradation VIII (to propanoate)	0.54	0.571	0.03319	TRUE
MetaCyc	L-phenylalanine degradation IV (mammalian, via side chain)	0.53	0.714	0.02364	TRUE
PlantCyc	superpathway of aspartate and asparagine biosynthesis	0.52	0.624	0.02851	TRUE
MetaCyc	benzoate fermentation (to acetate and cyclohexane carboxylate)	0.5	0.609	0.03113	TRUE

Sensitivity analysis of the reduced feature model (see Methods section for details and Supplementary Data 6 for results) demonstrated that out of the 22 metabolic pathways with a prediction value ≥ 0.5, only one metabolic pathway was misclassified, namely the MetaCyc listed superpathway of histidine, purine, and pyrimidine biosynthesis. Out of the 77 metabolic pathways with a prediction value < 0.5, 20.77% (16) were misclassified.

To verify the model, four pathways whose corresponding genes could be identified in the tomato genome were further subjected to in vivo analysis to show their existence in the tomato pericarp; two pathways corresponding to MetaCyc: the β-alanine degradation I, the L-tryptophan degradation VII (via indole-3-pyruvate); and two pathways corresponding to PlantCyc: the melibiose degradation and the β-alanine biosynthesis III pathways.

**Fig. 5:**
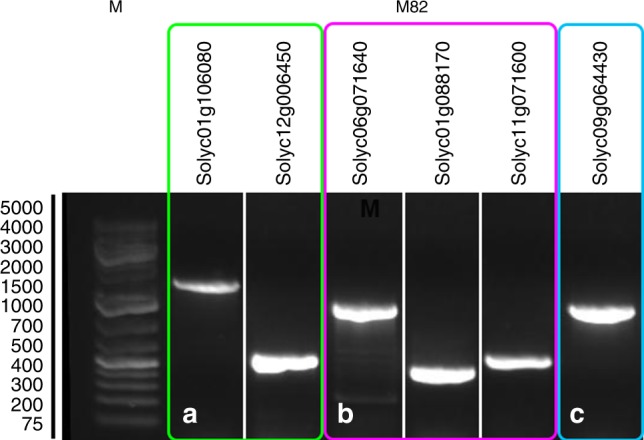
PCR validation of tomato genes. PCR amplification of tomato genes *Solyc01g10680, Solyc12g006450, Solyc06g071640, Solyc01g088170, Solyc11g071600, Solyc09g064430* from DNA extracted from tomato fruits. Amplicons are visible (M−1Kb + DNA ladder). Figure shows lanes spliced together corresponding to genes affliated with the same pathway—original gel can be viewed in Supplementary Fig. 3. **a** genes corresponding to the β-alanine degradation I pathway; **b** genes corresponding to the L-tryptophan degradation VII (via indole-3-pyrtuvate) MP; **c** gene corresponding to the β-alanine biosynthesis III pathway

The β-alanine degradation I is a two-step pathway, where β-alanine is catalyzed via β-alanine aminotransferase (EC 2.6.1.19) to 3-oxoproponate and then via malonate semialdehyde dehydrogenase (EC 1.2.1.18—also known as methylmalonate-semialdehyde dehydrogenase) to CO_2_. The first conversion also produces 2-oxoglutarate and L-glutamate, the second conversion produces an acetyl CoA NADH. In tomato *Solyc12g006450* codes for β-alanine aminotransferase and *Solyc01g106080* for malonate semialdehyde dehydrogenase. The presence of both genes was validated by performing PCR on DNA extracted from M82 tomatoes. A single amplicon was detected for both genes (Fig. 5a, Supplementary Fig. 3) and was confirmed via direct sequencing. The L-tryptophan degradation VII (via indole-3-pyruvate) is a three-step metabolic pathway, where tryptophan is converted into indole-3-pyruvate via tryptophan transaminase (EC 2.6.1.27 - *Solyc06g071640*—Supplementary Data 7), which is converted into indole-acetaldehyde, which is converted into indole-3-acetate. Both final steps can be catalyzed via indole-3-acetaldehyde oxidase / indolepyruvate decarboxylase (EC 1.2.3.7 / 4.1.1.74 - *Solyc01g088170 / Solyc11g071600*—Supplementary Data 7). Also here, the presence of all genes encoding enzymes that regulate the L-tryptophan degradation VII pathway was validated by performing PCR on DNA extracted from M82 tomatoes (Fig. 5b).

**Fig. 6:**
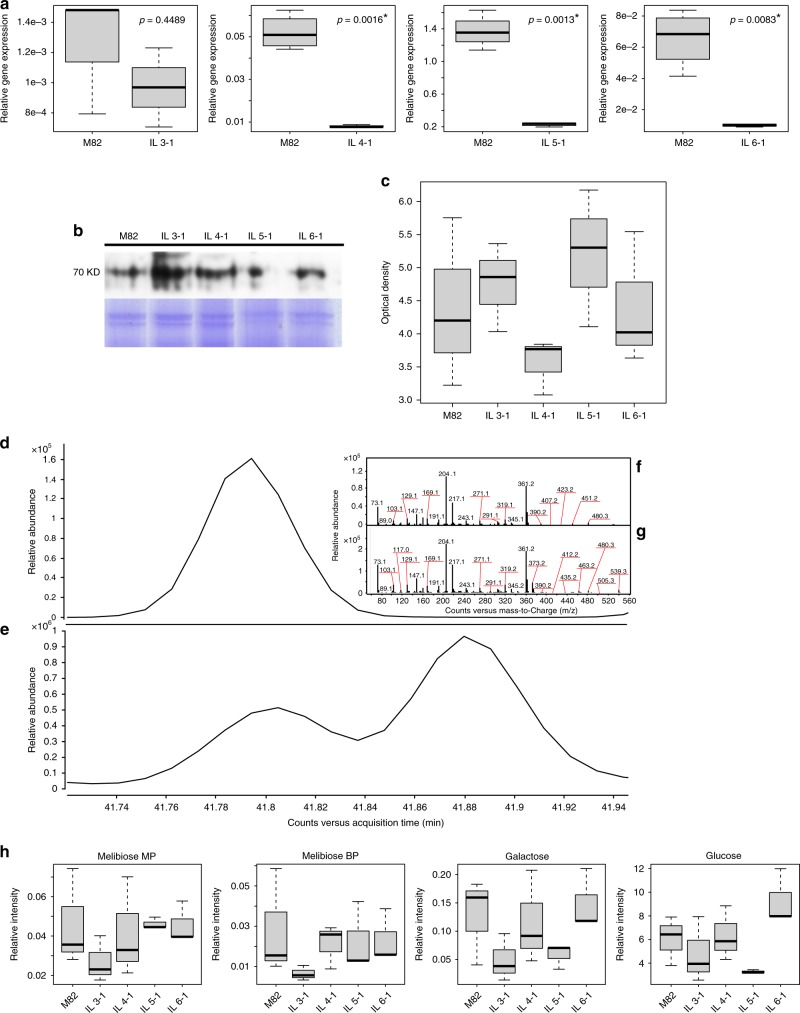
In vivo validation of the melibiose degradation pathway in the tomato pericarp. **a** Boxplot representation of quantitative analysis of transcripts with real-time RT-PCR performed for genes *Solyc03g019790* on introgression line (IL) 3-1, *Solyc04g008730* on IL 4-1, *Solyc05g013720* on IL 5-1, and *Solyc06g050130* on IL 6-1. The center lines represent the median; box limits represent upper and lower quartiles; whiskers represent 1.5 × interquartile range. The expression of each line was compared with M82 after normalization to *SGN-U314153*. The data represents the mean obtained for representative experiments from three independent biological replications. The *Student’s t-test* was applied to compare the relative expression levels. The values denoted by asterisk are significantly different (in which * indicates *p* < 0.05; ** indicates *p* < 0.01; and *** indicates *p* < 0.001). **b** Immunological analysis of α-galactosidase against corresponding barley antibodies. **c** Boxplot of colorimetric assay using p-nitrophenyl-α-D-galactopyranoside (pNPGal) as artificial substrate to test for α-galactosidase activity. The center line represents the median; box limits represent upper and lower quartiles; whiskers represent 1.5 × interquartile range. **d** Eluted melibiose standard chromatogram. **e** Tomato pericarp melibiose chromatogram. **f** Deconvoluted spectra of melibiose standard. **g** Deconvoluted spectra of melibiose in tomato pericarp. **h** Boxplot of quantitative analysis of melibiose main and byproduct, glucose, and galactose. Error bars represent standard deviation. The center lines represent the median; box limits represent upper and lower quartiles; whiskers represent 1.5 × interquartile range

The melibiose degradation is a single-step pathway, where melibiose is degraded via α-galactosidase (EC 3.2.1.22) to the sugars galactose and glucose^27^. Four loci associated with genes coding for α-galactosidase were detected within the tomato genome on chromosomes 3-6 (Supplementary Data 7)^28^. To further verify the existence of the pathways, in vivo assays were carried out on the transcript, enzymatic, and metabolite level, verifying the presence and activity of α-galactosidase regulating the melibiose degradation pathway. Quantitative RT-PCR showed that the expression levels were not significantly different (*p*-value = 0.4489) for *Solyc03g019790* on chromosome 3 between the parental line M82 and IL 3-1 (Fig. 6a, Supplementary Table 3). *Solyc04g008730* on chromosome 4, *Solyc05g013720* on chromosome 5, and *Solyc06g050130* on chromosome 6 all were shown to be differentially expressed on M82 and the respective ILs (Fig. 6a, Supplementary Table 3—respective *p*-values = 0.0016, 0.0013, 0.0083). To test for the presence of α-galactosidase, immunological analysis was performed against corresponding antibodies raised against α-galactosidase from barley^29^, revealing different amounts of the enzyme in M82 and the tested introgression lines (Fig. 6b). In order to test for α-galactosidase activity, aliquots of crude protein extract were subjected to a colorimetric assay using p-nitrophenyl-α-D-galactopyranoside (pNPGal) as artificial substrate. The analysis showed activity in all of the lines tested (Fig. 6c). Quantitative levels of melibiose, glucose, and galactose were also measured in the lines of interest. To the best of our knowledge, this is the first study reporting melibiose in the tomato pericarp. To verify the presence of melibiose in tomato, eluted melibiose standard (Fig. 6d) vs. putatively identified melibiose in the tomato pericarp (Fig. 6e) is presented, as well as their corresponding deconvoluted spectra (standard; Fig. 6f vs. sample; Fig. 6g). Quantitative analysis of melibiose main and byproducts, glucose, and galactose showed varying levels in M82 and the introgression lines (Fig. 6h).

The β-alanine biosynthesis III is one-step pathway, where L-aspartate is converted to β-alanine via aspartate 1-decarboxylase / L-tyrosine decarboxylase (EC 4.1.1.11/4.1.1.25 - *Solyc09g064430*). Also here we performed PCR on DNA extracted from M82 tomatoes to validat its presence Fig. 5c.

## Discussion

Understanding of the activity of metabolic pathways in the context of complex metabolic coordination^30^ is a key aspect in many domains, including agriculture (crop improvement) and health care (drug design). The constraint-based approach is the common (non-curated) method for proposing the existence of metabolic pathways in an organism; in this approach, genes regulating metabolic pathways are organized into genome-scale networks^6–8^, complemented by enrichment analysis based on expression data (gene ontology) for the identification of key metabolic processes. However, this approach ignores the post-regulatory mechanisms taking place between the genetic, enzymatic, and metabolic levels of the cell.

The approach demonstrated here is based on quantitative measurements of metabolites, and by that effectively accounts for post-transcriptional and post-translational events, circumventing the need for gene data integration. We showed that metabolic correlation-based networks incorporate more information about the cellular activity than has been attributed to them so far. In fact, our study shows that metabolic pathways are deeply embedded into metabolic CNs and shape their topological structure.

To detect metabolic pathways within metabolic CNs, network analysis was combined with ML techniques. In this analysis, 169 known metabolic pathways in tomato (positive instances) were mapped as subgraphs onto the three metabolite CNs of the pericarp of a tomato introgression line mapping population^10^. Additionally, 85 metabolic pathways unassociated with tomato from the MetaCyc collection as well as 85 random subsets of metabolites were added to the training set as negative instances. During the first trials of the study, initial ML models classified some tomato-unassociated pathways as tomato metabolic pathways, although their corresponding nodes in the CN did not correlate. We attribute this behavior to the fact that the initial ML models were trained using only tomato-associated metabolic pathways (positive instances from TomatoCyc) and tomato-unassociated metabolic pathways (negative instances from MetaCyc); thus, only a few negative instances contained uncorrelated metabolites within the studied CNs, not providing sufficient negative examples for the ML classifier to learn from. This observation led to the necessity to include also random subsets of metabolites as negative instances.

For each pathway (instance) a set of 148 (Supplementary Data 4) network-topological properties were computed for each season (Supplementary Data 1). In order to avoid overfitting and to identify the most contributing features, an InfoGain algorithm^26^ was applied, reducing the complete feature set to the 20 most relevant features (Fig. 3, Supplementary Table 2). The random forest model achieved an AUC of .923 (Fig. 2, Table 1) based on the top-20 chosen features. Notably, these features (Fig. 3) corresponded to the CN of season II, which had shown to be the most densely connected graph of the three networks and thus may incorporate more information in regard to metabolic pathways. This finding may be the outcome of meteorological changes between seasons impacting network topologies^11^, where season II may have presented favorable conditions with regard to pathway detection. In particular, features related to centrality measures and to node connectivity were identified in the reduced feature set. The high InfoGain of connectivity related properties indicated that nodes of pathways associated with tomato were more densely connected to each other than nodes of pathways not associated with tomato. The high InfoGain of centrality related features indicated that connections between nodes in different tomato pathways were stronger than connections between nodes in tomato unassociated pathways.

To predict the existence of previously unidentified pathways in tomato, a test set, composed of 33 plant metabolic pathways (PlantCyc) and 66 non-plant species metabolic pathways (MetaCyc), was generated. Similar to the pathways in the training set, each pathway in the test-set was mapped onto the three different CNs, followed by feature computation (Fig. 4). Based on the reduced feature-set, the existence of each of the 99 unidentified pathways was estimated by the trained random forest model. In total, 22 pathways that were previously not known as tomato pathways were here classified as such with a confidence level above 0.5 (Table 2, Supplementary Data 6). Sensitivity analysis confirmed all but one predicted pathway.

Limited in vivo analyses were performed on metabolic pathways with high prediction scores. Representative for MetaCyc, the β-alanine degradation I pathway and the L-tryptophan degradation VII (via indole-3-pyrtuvate) were chosen. The presence of all genes regulating both metabolic pathways were validated by performing PCR on DNA (Fig. 5a, b). The set of metabolites involved in the β-alanine degradation I and the L-tryptophan degradation VII metabolic pathways intersects with metabolites commonly reported in tomato in the current and other study. To date the β-alanine degradation I pathway has been associated with non-plant organisms, particularly in rat^31–33^. The named metabolic pathway is attributed with energy conservation in form of a CoA ester during the oxidation of an aldehyde^32^. The L-tryptophan VII pathway has been documented in non-pathogenic bacteria associated with plants^34^. Given that the set of metabolites and genes (and likely enzymes) is also present in tomato, it is probable to assume both metabolic pathways fulfill a similar purpose here.

Representative for PlantCyc, the β-alanine biosynthesis III and the top-scoring melibiose degradation pathway were analyzed. The β-alanine biosynthesis III is one-step pathway regulated by *Solyc09g064430*. In plants, only the aspartate 1-decarboxylation is associated with aspartate 1-decarboxylase. In Archaea also L-tyrosine decarboxylase has been shown to mediate the decarboxylation of aspartate^35^. Here, the presence of the gene encoding an L-tyrosine decarboxylase was validated via PCR performed on DNA extracted from M82 tomatoes (Fig. 5c). Nevertheless, the demonstration of the β-alanine biosynthesis III in tomato requires further research.

The melibiose degradation pathway is also a one-step metabolic pathway, where α-galactosidase cleaves melibiose into glucose and galactose. Note that only glucose and galactose were represented in the analyzed CNs. First, the transcript levels of four genes transcribing for α-galactosidase were tested, showing transcription in M82 and all corresponding ILs (Fig. 6a). Second, the presence of α-galactosidase was assayed applying immunological analysis, showing varying levels in the different lines tested (Fig. 6b). Third, α-galactosidase activity was tested using colorimetric tests. Results were indicative for activity in M82 and all corresponding lines (Fig. 6c). Finally, the presence of melibiose, glucose, and galactose were detected in the tomato pericarp (Fig. 6d–h). It is possible to claim that the mere presence of the abovementioned cellular compounds is not evidence of the melibiose degradation pathway, particularly since α-galactosidases catalyze the hydrolysis of various storage substances in plants. However, the melibiose degradation pathway is catalyzed only by α-galactosidase. This fact, coupled with our in vivo results, is substantial evidence of this metabolic pathway’s presence in the tomato pericarp. To the best of our knowledge, this is the first study to report the melibiose degradation pathway in tomato.

The actual power of the novel method for metabolic pathway detection presented here is revealed when placing the results in context of the initial datasets. Twenty years into metabolomics and tools for the definition of metabolites on an individual level are still lagging^36^. Commonly, the functionality of metabolites is determined based on their compound class affiliation rather than on the metabolite’s individual characteristics, exacerbated by enzyme promiscuity, cell compartmentation, and the complexity of metabolite networks^36^. The contextualizing of metabolic pathways into the CNs, as described in the current study, allowed to derive metabolite functionality with respect to metabolic pathways on an individual level. For instance, although all metabolites comprising the β-alanine degradation I pathway are present in tomato, this is the first study report its presence in tomato and in plants in general. Previous studies^10^ did not include melibiose in their respective datasets. The methodology described here was able to identify the melibiose degradation pathway although melibiose was not part of the initial (training) dataset. However, it is important to highlight that the approach presented here may be used to predict metabolic pathways but it cannot be used to predict differences in catalytic activity.

The usage of CNA combined with ML techniques will greatly contribute to metabolite pathway prediction and identification in incomplete datasets. In addition, the identification of metabolic pathways may be more accurate, as CNs are based on quantitative metabolic data taking into account all post-regulatory mechanisms occurring along the cellular machinery – a capability that is absent in the constraint-based pathway identification. Finally, as metabolic profiling can be performed independent of an annotated genome, the identification and prediction of metabolic pathways can be applied to virtually all organisms.

## Methods

### General statistics and reproducibility

To construct metabolite CNs the metabolic profiles of the tomato pericarp of an introgression line mapping population^24^ as generated for Schauer et al.^10^ were used. The dataset was composed of metabolite profiles of the central metabolism from three different harvesting seasons (field experiments) in three different years, hereinafter referred to as seasons I, II, and III. Each metabolite profile was based on 4 to 6 biological replicates. For each season a weighted, undirected metabolite CN was constructed as described by Toubiana et al.^11^. Network nodes represented metabolites and network links were weighted according to their Pearson correlation coefficient, allowing negative values. Spurious correlations, where *|r|* ≤ 0.3 and *p* ≥ 0.01, were removed (for details on how to generate metabolite CNs we refer the reader to Toubiana et al.^9^, where a pipeline for CN construction was suggested). For more details of network construction we refer the reader to the subsequent sections.

Metabolic pathway mapping and onto CNs was achieved with pathways from the PlantCyc and MetaCyc databases (see below for details). Feature computation was achieved with R code as provided in https://github.com/toubiana/CNA_combined_with_ML. The resulting feature-value datasets for positive and negative instances are supplied as Supplementary Data 1 and 2.

Statistical tests for validation of metabolic pathways was performed with *n* ≥ 3 biological replicates. A priori statistical tests were performed followed by adequate data transformation where necessary. Gene information for PCR performed on selected genes is provided in Supplementary Data 7 and Supplementay Table 4.

### Network construction

Metabolite CNs are represented as weighted networks *Gi* = (*V*_*i*_, *E*_*i*_, *w*), where *V*_*i*_ is the set of nodes corresponding to metabolites found in the dataset of season *i*, *E* is the set of links between them, and link weights (*w*:*E*→*R*) correspond to the Pearson correlation coefficient. In the rest of this paper we will use the terms nodes and metabolites interchangeably. The constructed CN for season I was composed of $|V_I| = 75$ nodes and $\left| {E_I} \right| = 473$ links connecting them; the CN for the season II was composed of $\left| {V_{II}} \right| = 75$ nodes and $\left| {E_{II}} \right| = 869$ links. The CN for season III was composed of $\left| {V_{III}} \right| = 78$ nodes and $\left| {E_{III}} \right| = 338$ links.

As a plant pathway reference, the PlantCyc database (http://www.plantcyc.org/) version PMN 10.0 was used, listing 1214 pathways, composed of 6200 reactions, involving 152,416 enzymes and 5138 compounds. For tomato pathways, the TomatoCyc database version 1.0 within PlantCyc was used, listing 589 pathways, composed of 3379 reactions, involving 7106 enzymes and 2557 compounds. Finally, the MetaCyc pathway database (http://metacyc.org/) version 20.0 was used, listing 2454 pathways from 2788 different organisms, composed of 13,533 reactions, involving 11,041 enzymes and 13,191 compounds. MetaCyc pathways that were also found in PlantCyc were regarded as a part of the PlantCyc pathway collection. In addition, random sets of two to 18 metabolites were generated, corresponding to the minimum and maximum length of coherent pathways identified in all three networks. Only metabolic pathways that that shared at least two compounds with all three CNs were relevant for subsequent analysis. Of the 3043 metabolic pathways, only 320 such pathways were identified.

### Feature engineering

Manual feature engineering is a laborious task, requiring detailed knowledge about the domain under investigation. Commonly, it is opted to produce a large number of features, which can be subsequently reduced via ML associated feature selection algorithms. Pathways from the aforementioned databases were mapped onto the networks by detecting metabolites within the pathway, which were also found in all three CNs. Network-based features were computed for each pathway as follows:

First, we used previously defined structural properties to quantify the importance of nodes and describe their location within the network: number of neighbors, weighted degree, closeness centrality, betweenness centrality, stress centrality, and clustering coefficient^19^. The edge betweenness centrality was used to quantify the importance of links. Structural properties for quantifying the relations between node pairs used in this study were: geodesic distance, Jaccard coefficient, preferential attachment score, and friends measure^37^. All of these properties were aggregated to produce the features of the pathways using the sum, the mean, and the three central moments.

Second, we applied various community detection algorithms^20^ on each CN and computed features based on the resulting communities (i.e., densely connected clusters of nodes). The set of communities is denoted as $C_i = \left\{ {C_i^1,C_i^2, \ldots ,C_i^k, \ldots } \right\}$ where *k* is the index of a community in CN of season *i*. A pathway *j* can be represented as a subset of metabolites in the CN of season *i*, denoted as $S_i^j \subseteq V_i$. Dispersion of metabolites across the various clusters may indicate the existence or absence of the respective chemical reactions. Therefore, the ratio of the metabolites of a pathway co-residing in the largest community $MAX_k\left\{ {\left| {C_i^k \cap S_i^j} \right|/\left| {S_i^j} \right|} \right\}$ is an important feature.

Next, we computed structural features from the neighborhoods of each pathway *j*. We denoted the neighborhood of the node *v* in the CN for season *i* as the following: ${\mathrm{\Gamma }}_i\left( v \right) = \left\{ {u:\left( {v,u} \right) \in E_i} \right\}$. Note that ${\mathrm{\Gamma }}_i\left( v \right)$ is the set of all metabolites that are significantly correlated with *v*. It is possible to compute various features from the neighborhoods of nodes in each pathway.

$${\bf{Intersection}}{\bf{:}}\ I_i^j = \left| {\mathop {\bigcap}\nolimits_{u \in S_i^j} {{\mathrm{\Gamma }}_i\left( u \right)} } \right|$$,

$${\mathbf{Union}}{\bf{:}}\ U_i^j = \left| {\mathop {\bigcup}\nolimits_{u \in S_i^j} {{\mathrm{\Gamma }}_i\left( u \right)} } \right|,$$

$${\bf{Distinct}}\,{\bf{neighborhoods}}{\bf{:}}\ D_i^j = \left| {\left\{ {u:\exists _{v \in S_i^j},u \in {\mathrm{\Gamma }}_i\left( v \right) \wedge \neg \exists _{v \ne q \in S_i^j},u \in {\mathrm{\Gamma }}_i\left( q \right)} \right\}} \right|$$, and

$${\bf{Mixed}}\,{\bf{neighborhoods}}{\bf{:}}\ M_i^j = U_i^j - I_i^j - D_i^j$$

The Distinct neighborhoods feature accounts for all nodes that are significantly correlated to exactly one metabolite within the pathway *j*. The Mixed neighborhoods feature accounts for all nodes that are significantly correlated to more than one metabolite within the pathway *j*, but not all of them. We note that these two features are reminiscent of the symmetric difference as defined in set theory. In fact, for two nodes, the Distinct neighborhoods feature is equal to the size of the symmetric difference of their neighborhoods. However, for a larger number of nodes both features are different from the symmetric difference.

Finally, metabolic pathways were mapped as subgraphs onto the different CNs. Two types of subgraphs were considered: conjunctive subgraphs and extended subgraphs. Conjunctive subgraphs included all nodes in $S_i^j$ and links between them, denoted as $SG_i^j = \left( {S_i^j,\left\{ {\left( {u,v} \right) \in E_i:u \in S \wedge v \in S} \right\},w} \right)$. Extended subgraphs included all nodes in $S_i^j$ as well as all of their neighbors, denoted as $ESG_i^j = \left( {V\prime ,E\prime ,w_i} \right)$, where $V\prime = \mathop {\bigcup}\nolimits_{v \in S_i^j} {{\mathrm{\Gamma }}_i\left( v \right)}$ and $E\prime = \left\{ {\left( {u,v} \right) \in E_i:u,v \in V\prime } \right\}$. Network features (diameter, diameter centrality, global clustering coefficient, assortativity, density) computed on these two types of subgraphs were used to describe the pathways. In addition, all features related to the centrality of nodes and links were computed on the conjunctive subgraph.

The complete list of 148 features and their verbal definitions can be found in Supplementary Data 4. The three CNs examined (corresponding to the three harvesting seasons I, II, and III) exhibited different topologies and thus, different feature vectors. These vectors were combined into a single feature vector comprised of 444 features. The actual numerical outcomes for all of the pathways examined for each season can be found in Supplementary Data 1. All of the features were computed using igraph^38^ and standard libraries in R^39^.

### Feature selection

The dataset we analyzed included 339 pathways for which 444 features were computed. A large number of features may impair the ability of an ML model to generalize beyond the data points used to produce it - a phenomenon known as overfitting. In an effort to avoid overfitting and identify the most contributing features, we selected the features with the highest information gain. This procedure reduces the entropy of the class variable after analyzing the value for the feature. For the current study, the top-20 ranked features (Fig. 3, Supplementary Data 3) were used to build the ML models for subsequent analysis (Supplementary Table 2). Feature reduction was performed using Weka^40^ version 3.6.11.

### ML model selection

In an effort to identify an ML algorithm that can be successfully applied to the pathway dataset generated, we tested several types of ML algorithms (e.g., decision trees, regression, Bayesian networks, etc.). ML algorithm tuning (a.k.a hyperparameter optimization) was performed applying a trial and error approach.

Given an instance whose class is unknown, a trained ML model assigns a probability of that instance being positive (a tomato pathway) or negative (a non-tomato pathway). If the probability of an instance having a positive class is above some predefined threshold, then the predicted class of that instance is positive. Standard performance metrics can be used to compare the predicted classes assigned to the pathways vs. their true classes, i.e., the true positive rate (TPR, recall), false positive rate (FPR), precision, and F-measure. In addition, the performance of ML models can be described by the receiver operating characteristic (ROC) curve, which is created by plotting the TPR as a function of the FPR at different threshold levels. The AUC under the ROC curve of ‘1’ indicates a perfect classifier. The AUC is often used as the pivotal measure, because it does not require specifying the threshold and it is independent of the proportion of positive and negative instances in the dataset.

### In silico model validation

There are several procedures that can be used to evaluate the ability of an ML model to predict the class of previously unseen instances. The most popular method is k-fold cross-validation, where the dataset is divided into k equal (equal number of instances) subsets. Each subset is then removed from the dataset in its turn. An ML model is trained based on the remaining subsets. The trained model is applied on every instance in the removed subset, and the predicted class is recorded. Eventually after k iterations all instances in the dataset will be assigned a predicted class vs. their original true class. Cross-validation is typically used to prove the stability of a given ML algorithm and assess whether or not the trained model is prone to overfitting. On one hand, a larger number of folds results in a larger number of instances in the training set during each iteration and consequently renders more accurate models. On the other hand, a larger k requires training more ML models during the evaluation, which increases the computational resources required.

Due to the large number of ML algorithms evaluated, 10-fold cross-validation was used to select the best ML algorithm for the current study. Once the best ML algorithm was chosen, we increased the number of folds to the maximal possible value (339 pathways in our case) in order to obtain the most accurate in silico evaluation results. This special case of k-fold cross validation is known as leave-one-out cross-validation (LOOCV)^41^.

All ML modeling and testing was performed using Weka^40^ software, version 3.6.11. For the current study, the best model was achieved using the random forest algorithm and an equal distribution between MetaCyc and randomly engineered pathways (Fig. 2, Table 1). The random forest model was run with 100 trees, each constructed while considering nine random features, and an out-of-bag error of 0.1711.

### Balanced training set and negative sampling

Out of the 589 TomatoCyc pathways investigated in this study, 169 pathways were identified within each of the three CNs. These pathways were used as the positive instances of the training set. The total number of MetaCyc pathways that were not represented in PlantCyc and could be selected as the negative instances was 151. Using the 169 TomatoCyc pathways as the positive instances and the 151 MetaCyc pathways as the negative instances resulted in inadequate performance of the ML models. In particular, sets of metabolites consisting of disconnected nodes were rated disproportionally high. ML models perform best when they are trained using a balanced training set where there is an equal number of positive and negative instances^42^. In order to tackle this bias we employed the random sampling methodology by adding non-pathways (i.e., randomly generated sets of 2-18 metabolites) as negative instances to the dataset^43^. Therefore, all of the positive instances were used for training, along with 85 randomly selected MetaCyc pathways and the same number of randomly selected non-pathways. In total, 170 negative instances were produced (Supplementary Data 2).

### Sensitivity analysis

Sensitivity analysis was performed based on the final model, where a subset with 80% of the training set instances were randomly chosen to recreate a model with identical settings. After each model generation the test set instances were subjected to prediction. This analysis was performed with 100 iterations, after which the corresponding average and variance values were computed.

### Real-time quantitative RT-PCR analysis

M82 tomato fruits were freeze lyophilized and grounded to a fine powder prior to extraction. Total genomic DNA was extracted with Hexadecyl trimethyl-ammonium bromide (CTAB)^44^. Fragments corresponding to specific genes regions were PCR amplified using CloneAmp HiFi PCR Premix (Katara) with the primers shown in supplementary Table 4. Each 25 µL reaction volume contained 12.5 µL of 2 × master mix, 1 µL of 10 µM primer, 2 µL of cDNA aliquot, and 9.5 µL ddH2O. The reactions were conducted in a thermal cycler with initial denaturation at 94 °C for 5 min, followed by 35 cycles of 94 °C for 15 s, 60 °C for 15 s, 72 °C for 30 s and then a final extension at 72 °C for 5 min.

For relative quantitative RT-PCR, total RNA was extracted from the mesocarp tissue (excluding the seed) from the ILs of interest and M82 using an Aurum Total RNA Kit according to the manufacturer’s instructions (Bio-Rad, http://www.bio-rad.com/). First-strand cDNA was synthesized in a 10 μL volume, containing 350 ng of plant total RNA by using an iScript cDNA Synthesis Kit (Bio-Rad). The reaction was carried out using 1:10 dilutions of cDNA. PCR was hot started at 95 °C and carried out for 40 cycles composed of 95 °C for 20 s, 65 °C for 20 s, and 72 °C for 30 s. Amplification was monitored in real-time using an iCycler IQ multicolor real time PCR Detection System (Bio-Rad). The list of primers (Supplementary Table 4) was designed for exon junctions by the Primer3 program (http://bioinfo.ut.ee/primer3-0.4.0/primer3). The relative contents of transcripts was determined by the 2–ΔΔCt method^45,46^ based on the normalization of expression data with regard to the expression of one reference gene. The reference genes were *SGN-U314153* and *SGN-U316474*, characterized by constitutive expression^47^. The differences of Ct (ΔCt) between the control and target were compared.

### Immunological analysis of α-galactosidase

Plant tissues were frozen in liquid N_2_ and grinded in extraction buffer [HEPES 50 mM, NaCl 100 mM, KCl 10 mM, 0.4 M sucrose, PMSF 1 mM, and protease inhibitor 1% (v/v)]. The homogenate was centrifuged 20,000 × *g* for 10 minutes at 4 °C, and the protein concentration was determined in the supernatant using a Bradford assay^48^. Proteins (20–30 µg) were separated by SDS-PAGE, and transferred to a polyvinylidene difluoride membrane (Bio-Rad, Hercules, CA). Blotting and incubation with a primary antibody raised against alpha-galactosidase from barley were performed as described by Chrost and Krupinska^29^. As a secondary antibody, a peroxidase-coupled anti-rabbit serum was used for visualization of immunoreactive protein bands.

### α-galactosidase activity assay

Enzyme extraction and assay were performed as previously described in Sozzi et al.^49^ with certain modifications. Mesocarp tissue (excluding the seed) from 5-10 fully mature tomato fruits weighing 50 g was cut into small pieces and suspended with 50 mL 1 M NaCl (pH was adjusted to six). The suspension was homogenized with glass beads at 4 °C for a period of 30 s. The resultant suspension was stirred for one hour at 4 °C, filtered through cheesecloth, and centrifuged at 12,000 × *g* for 20 min. The supernatant fraction was loaded onto PD SpinTrap G-25 columns (www.gelifesciences.com, GE Healthcare UK Ltd Buckinghamshire, UK), pre-equilibrated with 20 mM sodium acetate/acetic acid buffer (pH 4.75). The desalted protein was then eluted with the same buffer (20 mM sodium acetate/acetic acid) and used immediately for enzyme assaying.

In order to test for α-galactosidase activity, aliquots of crude protein extract were assayed as previously described^50^ using p-nitrophenyl-α-D-galactopyranoside (pNPGal) as substrate. The assay mixture, composed of—40 μL of 26 mM pNPGal, 50 µL of 100 mM acetate buffer (pH 4.5), and 40 µL of 0.2% BSA, was pre-incubated at 37 °C for two minutes, and the enzymatic reaction was initiated by the addition of 20 µL of the crude extract. Following 15min of incubation at 37 °C, the enzymatic reaction was terminated by adding 100 µL of 0.4 M Na_2_CO_3_, and the released yellow colored p-nitrophenol was determined spectrophotometrically at 410 nm. A blank solution absent the protein was run concurrently, and the appropriate correction was made.

### Metabolite extraction and quantification

Frozen pericarp tissue powder was extracted in chloroform-methanol, and metabolites were quantified by gas chromatography-mass spectrometry (GC-MS) following a procedure optimized for tomato tissue^51^. Pure standard of melibiose (purchased from Sigma) was diluted in methanol and run in different quantities to build calibration curves. In the standard, two peaks were identified (1MEOX) (8TMS) main-product and by-product (C37H89NO11Si8) MW 948 RI 2837 and 2868 by library RT 41.8 and 42.1 min. Extract sample (300 µL) was injected (1 µL) with and without spiked-in standard. Identification and annotation of melibiose was achieved based on comparison to an authentic standard. In addition, control samples with spiked-in non-labeled standards were also used to confirm coelution. Metabolite identity was further matched against publically available databases (the Golm Metabolome Database for GC-MS reference data:^52^ http://gmd.mpimp-golm.mpg.de/. A similar approach was followed for galactose and glucose.

### Reporting summary

Further information on research design is available in the Nature Research Reporting Summary linked to this article.

## Supplementary information

Supplementary Information

Supplementary Data 1

Supplementary Data 2

Supplementary Data 3

Supplementary Data 4

Supplementary Data 5

Supplementary Data 6

Supplementary Data 7

Description of Supplementary Data

Reporting Summary

## Data Availability

The datasets generated during the current study are available as Supplementary Data files (Supplementary Data 1-7).

## Code availability

Source code used to compute features of metabolic pathways within correlation networks was deposited on github: https://github.com/toubiana/CNA_combined_with_ML.
