# Supplementary material for: Combined network analysis and machine learning allows the prediction of metabolic pathways from tomato metabolomics data
Source: Commun Biol. 2019 Jun 18;2:214. doi: 10.1038/s42003-019-0440-4 (PMC6581905; doi:10.1038/s42003-019-0440-4)
Supplement: Supplementary file 1 — Supplementary Information [file 42003_2019_440_MOESM1_ESM.pdf]

Supplementary Figures

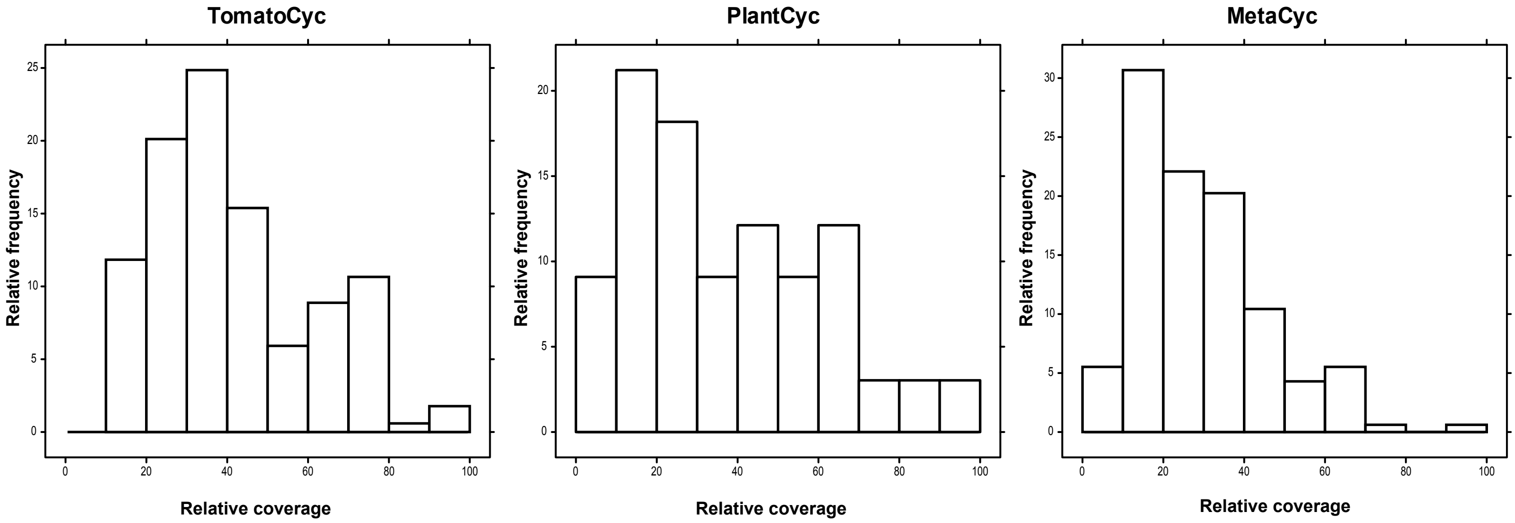

Supplementary Figure 1: Pathway coverage within the tomato metabolite CNs

The histogram shows the distribution of the relative portion of the metabolites of the different MPs mapped to the CNs. The metabolite sets of the three harvesting seasons were intersected and only pathways that could be allocated to the intersection were chosen (partial coverage with at least two metabolites was permitted). Out of the 589 pathways listed in the TomatoCyc repository, 169 could be found within the intersection; 33 of the plant pathways, which were not listed for the tomato could be found in the PlantCyc repository. Out of the 2,454 pathways listed in the MetaCyc database, 151 non-plant pathways demonstrated partial to full coverage within the metabolite intersection.

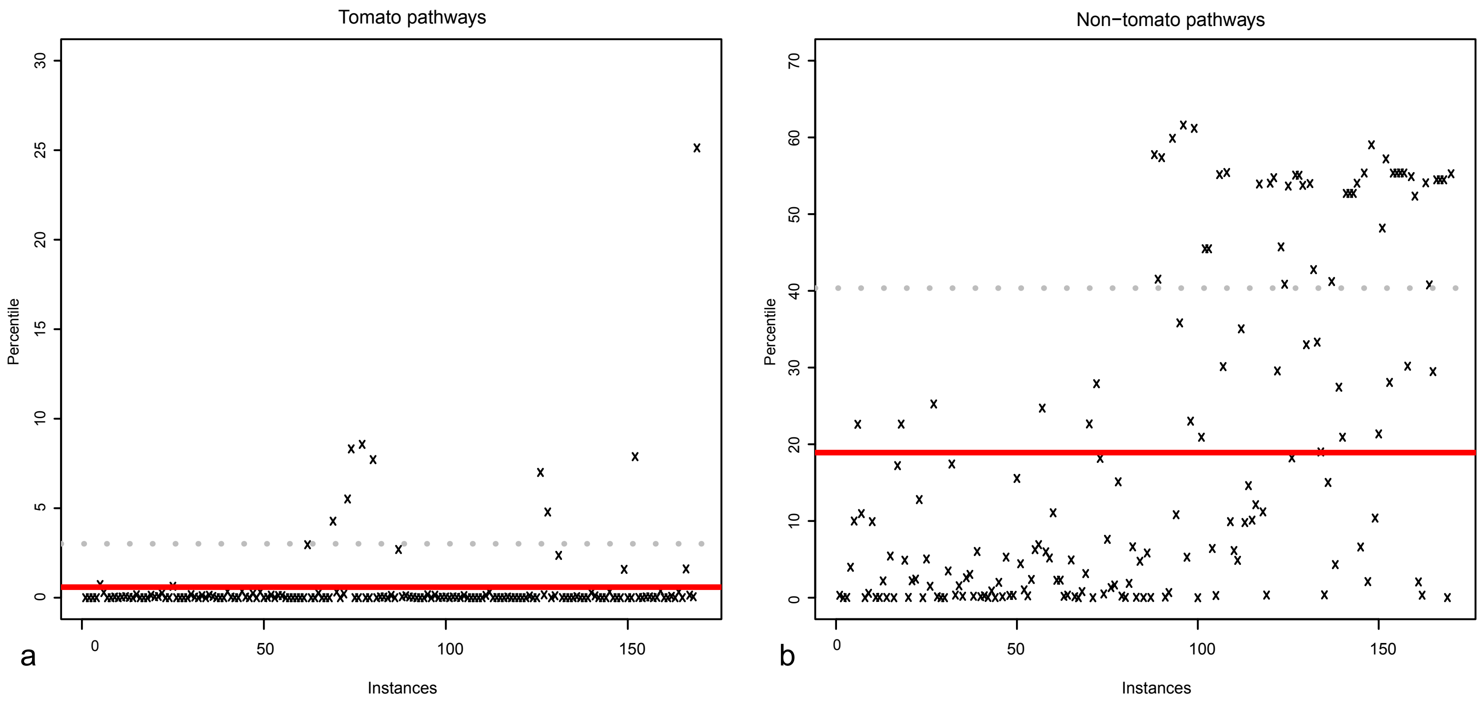

Supplementary Figure 2: Leave-one-out cross-validation (LOOCV)

The figure depicts the results of the leave-one-out cross-validation, where the predictive values and their corresponding rankings vs. ~1.7e+07 random pathways of 169 tomato pathways (a – positive instances) compared to 170 MetaCyc and random pathways (b – negative instances) are shown. The red horizontal lines illustrate the respective means, and the grey dashed line represents the standard deviation.

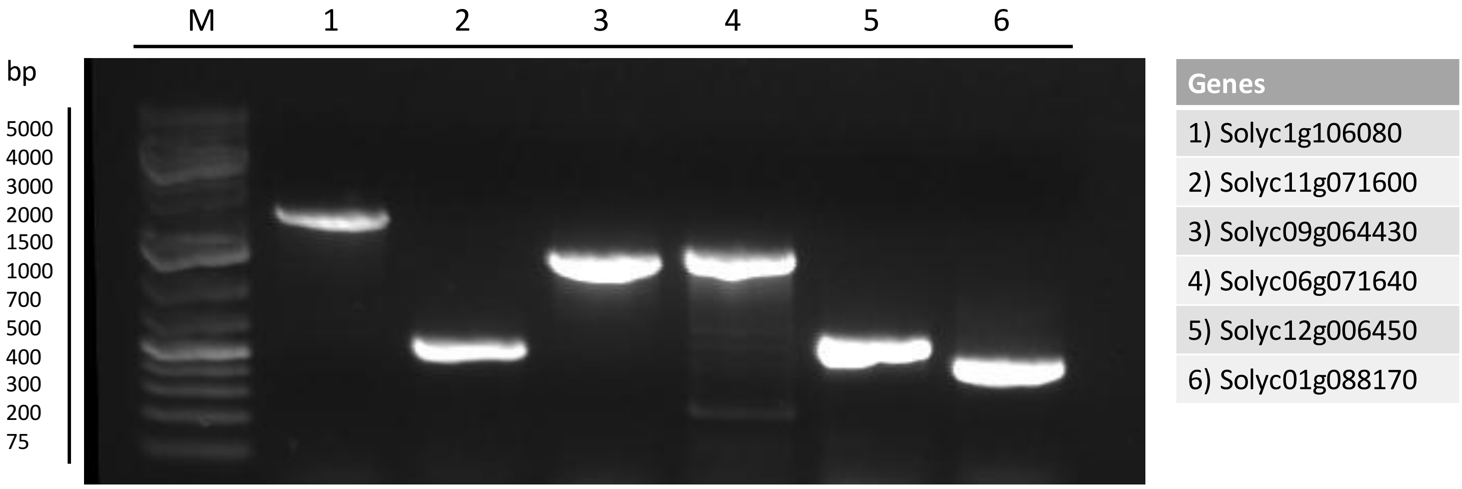

Supplementary Figure 3: Original PCR validation gel of tomato genes

Original PCR validation gel corresponding to Figure 5.

Supplementary Tables

Supplementary Table 1: Random forest model confusion matrix

	positive class	negative class
positive class	147	22
negative class	33	137

Supplementary Table 2: Top 20 seeded features

Feature	Season	Definition	Mathematical moment
Skewness absolute weighted degree of graph	II	The absolute weighted node degree quantifies the absolute weight of all links incident on a node. In case of correlation-based networks the weight of an link corresponds to the absolute correlation coefficient. Here it denotes all links within the subgraph and the links linking the nodes of the subgraph to the remaining nodes of the graph	skewness
Kurtosis absolute weighted degree of graph	II		kurtosis
Absolute weighted degree of graph	II		---
Standard deviation of absolute weighted degree of graph	II		standard deviation
Total absolute weighted degree of graph	II		Accumulative absolute weighted degree
Weighted standard deviation node betweenness centrality of subgraph	II	The weighted node betweenness centrality of a node *i* is given by the the number of weighted geodesic distances between any two nodes that contain node *i* – here delimited to the subgraph	Standard deviation
Edge number of graph	II	Total amount of links incident on adjacent nodes of subgraph including links to the nodes of the remaining graph	---
Edge betweenness community of subgraph	II	The edge betweenness community detecting algorithm is based on the edge betweenness centrality property. It applies a hierarchical decomposition process in which links are removed based on their betweenness score. Communities are build based on the idea that links connecting different communities are more likely to be contained as multiple shortest paths. Here a feature is constructed given by the ratio of how many nodes of the subgraph co-reside in the largest community as opposed to nodes that are located within other communities.	---
Mixed neighborhoods	II	The mixed neighborhoods feature accounts for all nodes that are	---

		significantly correlated to more than one metabolite within a pathway, but not all of them (see definition in Materials and Methods for more details)	
Union of neighborhoods	II	The union of neighborhoods features quantifies how many nodes/friends of order one all nodes within the subgraph have in total, counting each friend only once in the background of the entire graph and excluding friendship to each other (see definition in Materials and Methods for more details)	---
Total weighted degree of graph	II	The weighted degree quantifies the weight of all links incident on a node. In case of correlation-based networks the weight of a link corresponds to the correlation coefficient. Here it denotes all links within the subgraph and the links linking the nodes of the subgraph to the remaining nodes of the graph	Accumulative weighted degree
Weighted average closeness centrality of subgraph	II	The weighted closeness centrality is the reciprocal of the weighted average path length between a given node *i* and all other nodes in a given connected graph. Here, the closeness centrality was measured for every node in the subgraph.	Average
Average closeness centrality of subgraph	III	The closeness centrality is the reciprocal of the average path length between a given node *i* and all other nodes in a given connected graph. Here, the closeness centrality was measured for every node in the subgraph.	Average
Density of subgraph	I	The density of a graph is the number of links over the number of possible links – here delimited to the subgraph	---
Average closeness centrality of subgraph	I	See above	Average
Weighted average closeness centrality of subgraph	I	See above	Average
Weighted standard deviation local clustering coefficient of subgraph within graph	III	The local clustering coefficient of a node *i* is the proportion of existing links from all possible links between the neighbors of *i*, taking into account the weight of edges. It quantifies how close the subnetwork induced by *i* and its adjacent nodes is from a clique. Here, the local clustering coefficient is estimated for all nodes in the subgraph in the background of the entire graph.	Standard deviation
Leading eigenvector community of subgraph	I	The leading eigenvector community detecting algorithm applies a top-down hierarchical approach that optimizes the modularity function. In each step the graph is split into two parts in a way that	

		separation yields a significant increase in modularity. The split is performed by determining the leading eigenvector of the so-called modularity matrix. Here a feature is constructed given by the the ratio of how many nodes of the subgraph co-reside in the largest community as opposed to nodes that are located within other communities.	
Average closeness centrality of subgraph	II	See above	Average
Average weighted degree of graph	II	See above	Average

Supplementary Table 3: Quantitative analysis of transcripts by real-time RT-PCR was performed for M82, and introgression lines 3-1, 4-1, 5-1, 6-1, 12-1, 12-2 and 12-1-1. The expression of each line was compared with M82 after normalization to *SGN-U314153*. The data represent mean obtained from representative experiment from three independent biological replications. The values denoted by asterisk are significantly different, in which * indicates $p < 0.05$, ** indicates $p < 0.01$ and *** indicates $p < 0.001$.

	Solyc03g019790	*Solyc04g008730*	*Solyc05g013720*	*Solyc06g050130.2*	*Solyc12g006450.1*
M82	1±0.31	41.65±6.50	489.71±117.26	2.34±0.65	540.61±76.36
3_1	0.77±0.30				
4_1		6.44±0.53***			
5_1			127.51±20.36***		
6_1				0.44±0.22**	
12_1					41.13±7.48***
12_2					93.35±23.77***
12_1_1					109.79±15.73***

Supplementary Table 4: Sequence of primers

Gene ID	Primer Sequence
Solyc03g019790	5’AGACTCGCAGGGAAATATGGTTG3’<br>5’GTTTTTGCGTCTTGTTCTTCGTGT3’
Solyc04g008730	5’CGATTTCAAAGGCTCCCCTTATC3’<br>5’CCGATATCATCCCAGTTTGCTGT3’
Solyc05g013720	5’ACTGGATACCACTACGGGCAAAG3’<br>5’CACAAAATCAACACCCCAATCAG3’
Solyc06g050130.2	5’AGACTCGCAGGGAAATATGGTTG3’<br>5’GTTTTTGCGTCTTGTTCTTCGTGT3’
Solyc12g006450.1	5’ATCGCGGATGAGGTTATATGTGG3’<br>5’TGTACCGAGCTTGTTGCTTTGAG3’
SGN-U314153	5’GGTTTCTCAAGGTGTGGGAGAAG3’<br>5’TCCGATGTATAAGTGCTGGTGTGA3’
SGN-U316474	5’TGTTGGTGCACAAAAAGCATCTC3’<br>5’TGTCAACATCGAGGTAATGCACA3’
