# Supplementary material for: Combined network analysis and machine learning allows the prediction of metabolic pathways from tomato metabolomics data
Source: Commun Biol. 2019 Jun 18;2:214. doi: 10.1038/s42003-019-0440-4 (PMC6581905; doi:10.1038/s42003-019-0440-4)
Supplement: Supplementary file 9 — Description of Supplementary Data [file 42003_2019_440_MOESM9_ESM.pdf]

Description of Additional Supplementary Files

File Name: Supplementary Data 1
Description: **Training set pathways and computed features**
Training data set for machine-learning model generation

File Name: Supplementary Data 2
Description: **Test set pathways and computed features**
Test data set for testing machine-learning model

File Name: Supplementary Data 3
Description: **Feature ranking**
933 List of features sorted in descending order according to feature ranking

File Name: Supplementary Data 4
Description: **Feature list and description**
Verbal description of features used in machine-learning model

File Name: Supplementary Data 5
Description: **Leave-one-out cross-validation ranking of training set instances**
Values generated via leave-one-out cross-validation for each training set instance

File Name: Supplementary Data 6
Description: **Predictions of test set instances**
Prediction values for each test set instance

File Name: Supplementary Data 7
Description: **Details of candidate genes depicted in Figure 4**
Sequences and details of genes tested via PCR
